# How scientists use social media to communicate their research

**DOI:** 10.1186/1479-5876-9-199

**Published:** 2011-11-15

**Authors:** Laura Van Eperen, Francesco M Marincola

**Affiliations:** 1 Van Eperen & Company, Strategic Communications Consulting, Bethesda, MD 20817, USA; 2 Infectious Disease and Immunogenetics Section, Department of Transfusion Medicine Clinical Center and trans-NIH center for Human Immunology, National Institutes of Health Bethesda, Maryland, USA

## Abstract

Millions of people all over the world are constantly sharing an extremely wide range of fascinating, quirky, funny, irrelevant and important content all at once. Even scientists are no strangers to this trend. Social media has enabled them to communicate their research quickly and efficiently throughout each corner of the world. But which social media platforms are they using to communicate this research and how are they using them? One thing is clear: the range of social media platforms that scientists are using is relatively vast and dependent on discipline and sentiment. While the future of social media is unknown, a combination of educated speculation and persuasive fact points to the industry's continual growth and influence. Thus, is that not only are scientists utilizing social media to communicate their research, they must. The ability to communicate to the masses via social media is critical to the distribution of scientific information amongst professionals in the field and to the general population.

## How Scientists Use Social Media to Communicate Their Research

On any given day, 50% of Facebook's 500-million-plus users log-on to the social networking site. The average user is connected to 80 community pages, groups and events, and creates about 90 pieces of content for posting each month [1]. Meanwhile, Twitter's 200 million registered users will produce 110 million tweets per day on topics ranging from CNN's breaking news to celebrity gossip and more [2]. Factoring in the social communities of YouTube, LinkedIn and Ted.Com equates the masses. The bottom line is that millions of people all over the world are constantly sharing an extremely wide range of fascinating, quirky, funny, irrelevant and important content all at once. Even scientists are no strangers to this trend. Social media has enabled them to communicate their research quickly and efficiently throughout each corner of the world. But which social media platforms are they using to communicate this research and how are they using them?

One thing is clear: the range of social media platforms that scientists are using is relatively vast and dependent on discipline and sentiments, particularly when it comes to mainstream social networking sites such as Twitter and Facebook. In 2007, BioInformatics LLC conducted a survey with regard to scientists and social messaging:

• 77% of life scientists participated in some type of social media;

• 50% viewed blogs, discussion groups, online communities, and social networking as beneficial to sharing ideas with colleagues;

• 85% saw social media affecting their decision-making;

• Discussion groups and message boards were still the most-used types of sites, but online communities were gaining fast.

• User-generated content is not completely trusted for product information, but it is more trusted than information in printed trade magazines, editorial web sites, or online portals [3].

While many expect scientists to use social media like most other professionals, the diverse sites they choose to use and their varied skepticism of mainstream social media might come as a surprise. Many scientists perceive Facebook and Twitter, for example, as unprofessional platforms that may compromise or threaten years of life-changing research. As Brian Krueger, founder of the blogging and social network site LabSpaces.net, puts it, "Social networks first began to take off in the late 90 s, but the emergence of Facebook and Myspace in 2004 set a new trend for internet use. Although these sites have their merits, they don't provide an environment conducive to productivity. LabSpaces began as my desire to provide a productive social network for science by creating a website to attract a diverse set of researchers for the sole purpose of increasing communication and collaboration in the sciences." [4] LabSpaces is one of many social networking platforms utilized by niche scientific professionals who appreciate the power of communicating to the masses, but want to do so inside the walls of a gated web community. For example, Surgytec is a social networking platform for MDs and PhDs in the medical sciences. Started by HPJ Stevens, MD, PhD, a plastic surgeon from Rotterdam, The Netherlands, Surgytec operates globally, providing an integrated platform of videos, e-learning courses, discussion rooms, and blog posts. It allows its community of the best medical practitioners and academic peers to collaborate and share findings on topics ranging from Early Detection and Diagnosis of Lung Cancer and Immune Circuitry to the use of medical serums, such as Golgi protein 73 (GOLPH2), as markers for hepatocellular abnormal cell growth in the body [5].

And it's not only individual scientific professionals who have averted main stream social media and launched "boutique" social networks. Nature Publishing Group founded Sciatble, a platform for scientists, teachers, researchers, and students to connect through group discussion boards, posting of articles, and e-trainings. Focused on genetics and cell biology, the site spotlights topics such as the ability of enzymes to perform various processes related to DNA sequences. The site's list of sponsors is admirable, as it boasts high-profile corporate partners such as Intel and Roche Applied Science, a division of Roche Diagnostics [6].

Some scientists, however, have a soft spot for Twitter, Facebook, YouTube, and other such social networking sites that naysayers might consider trendy and wasteful. Stephen Hawking, for example, is an avid user of Twitter, utilizing the 140-character limit messaging system on a weekly basis. Though his "tweets" aren't always related to scientific research, he is integrating himself into a vast community worldwide, and exposing parts of his authentic personality to each of his thousands of followers [7]. The hope, then, is that his scientific-based messages are "re-tweeted" and shared by these loyal followers, who feel they have come to know him as a person in addition to a brilliant scientist. Likewise, Richard Dawkins, a famous British ethologist and evolutionary biologist, has two Facebook pages, one for Richard Dawkins the individual and one for his foundation, The Richard Dawkins Foundation for Reason and Science. With its mission to "Support scientific education, critical thinking and evidence-based understanding of the natural world, in the quest to overcome religious fundamentalism, superstition, intolerance and human suffering," the Dawkins Foundation's Facebook page and its 250,000 fans broadcast a sea of messages, such as links to live video discussions on the Kalam Cosmological Argument or articles on the evolution of man.

Similar to Sciatble, Twitter and Facebook are pillars of communications strategies for major scientific corporations. NASA Connect is the organization's web page focused exclusively on social media. Under the sub-heading "Connect with NASA on Social Networking Sites" are icons for nearly every major social networking site in existence, including Twitter, Facebook, MySpace, Gowalla, and YouTube. NASA even created its own "Tweetup group," which invites a portion of its 956,073 Twitter followers to go behind-the-scenes at NASA facilities and events to speak with scientists, engineers, astronauts, and managers. NASA's Jet Propulsion Laboratory hosted the first NASA Tweetup on Jan. 21, 2009. The next Tweetup is scheduled to take place on Thursday and Friday, April 28 and 29. NASA will invite 150 of its Twitter followers to the Kennedy Space Center in Florida for the launch of the Space shuttle Endeavour, Mission STS-134, and to speak with fellow "Tweeps" and NASA personnel[8].

Likewise, the National Geographic Society has grown its Facebook community from just under 2 million fans to more than 7 million fans in the span of a few short months. With a VP of Social Media within its ranks, the 123-year-old organization is dedicated to scientific outreach on a variety of community-based websites. Its daily messaging disseminates scientific information on the more than 7,000 islands in the vast archipelago east of Vietnam and the shrouded icy fog that turns to precipitation nightly on planet Mars. Accompanied by its world-renowned photography, the average post on the Society's Facebook page receives more than 3,000 "likes," indicating that its fan base is, indeed, absorbing the information provided. National Geographic's Facebook presence is also divisional, with individual pages dedicated to National Geographic Magazine, National Geographic's Global Action Atlas, Nat Geo Channel, and National Geographic Education[9].

Even organizations that we might not initially associate with science are seeing the grave importance of using social media to communicate scientific research. Internet search giant Google, and its philanthropic arm, Google.org, communicated its own motivators behind using social media for scientific communication: "In an effort to foster a more open, transparent and accessible scientific dialogue, we've started a new effort aimed at inspiring pioneering use of technology, new media and computational thinking in the communication of science to diverse audiences. Initially, we'll focus on communicating the science on climate change."

While the future of social media is unknown, a combination of educated speculation and persuasive fact points to the industry's continual growth and influence. Although the social media space started with only a small group of constituents, it has grown into a multi-billion dollar industry with rapid expansion to mobile devices. Supporting notions of social media's power and prevalence, President Barack Obama chose to make a policy speech earlier this year about the economy at none other than Facebook's headquarters [10]. Likewise, when Egypt's interim Prime Minister Ahmed Shafiq stepped down from his post, the announcement was initially made by Egypt's new ruler, the Supreme Council of the Armed Forces, on its official Facebook page, allowing for immediate communication to its more than 700,000 "friends."[11] When both the record-setting 8.9 magnitude earthquake and horrific tsunami hit Japan last March, millions rushed to social media sites to post news about loved ones, share photos and video footage, and even donate funds for relief efforts. Google launched a "Person Finder" web app to link victims with family members, and more than 7,000 records were entered on the actual day of the earthquake, March 11. By the very same afternoon, 9,000 earthquake-related videos and 7,000 tsunami-related videos had been uploaded to YouTube [12].

Propelled by ground-breaking research, political unrest, and extreme natural disasters occurring worldwide, the popularity of the social media phenomenon is not waning - it is exploding. In short order, it has gone from functioning as a powerful influence over current events to a phenomenon that serves as a vital communications tool used for survival. The conclusion, thus, is that not only are scientists utilizing social media to communicate their research, they must. Whether it is within the cyber walls of a prestigious one-off or on the same platforms being used by 13-year-olds, the ability to communicate to the masses via social media is critical to the distribution of scientific information amongst professionals in the field and to the general population. There was a time when "social media" was considered superfluous, merely a tool to distract ourselves from real-time events and discussions. We must move past such stigmatisms and recognize social media's power in communicating advancements in the scientific field by acknowledging that successful communication can only be achieved by employing the channels in which the general public is currently engaged.
